# Kanglemycin A Can Overcome Rifamycin Resistance Caused by ADP-Ribosylation by Arr Protein

**DOI:** 10.1128/AAC.00864-21

**Published:** 2021-11-17

**Authors:** John Harbottle, Hamed Mosaei, Nicholas Allenby, Nikolay Zenkin

**Affiliations:** a Centre for Bacterial Cell Biology, Biosciences Institute, Faculty of Medical Sciences, Newcastle University, Newcastle upon Tyne, United Kingdom; b Odyssey Newcastle, The Biosphere, Draymans Way, Newcastle upon Tyne, United Kingdom

**Keywords:** ADP-ribosylation, Arr, *Mycobacterium*, RNA polymerase, kanglemycin, rifampicin, rifamycin

## Abstract

Rifamycins, such as rifampicin (Rif), are potent inhibitors of bacterial RNA polymerase (RNAP) and are widely used antibiotics. Rifamycin resistance is usually associated with mutations in RNAP that preclude rifamycin binding. However, some bacteria have a type of ADP-ribosyl transferases, Arr, which ADP-ribosylate rifamycin molecules, thus inactivating their antimicrobial activity. Here, we directly show that ADP-ribosylation abolishes inhibition of transcription by rifampicin, the most widely used rifamycin antibiotic. We also show that a natural rifamycin, kanglemycin A (KglA), which has a unique sugar moiety at the *ansa* chain close to the Arr modification site, does not bind to Arr from Mycobacterium smegmatis and thus is not susceptible to inactivation. We, found, however, that kanglemycin A can still be ADP-ribosylated by the Arr of an emerging pathogen, Mycobacterium abscessus. Interestingly, the only part of Arr that exhibits no homology between the species is the part that sterically clashes with the sugar moiety of kanglemycin A in M. smegmatis Arr. This suggests that M. abscessus has encountered KglA or rifamycin with a similar sugar modification in the course of evolution. The results show that KglA could be an effective antimicrobial against some of the Arr-encoding bacteria.

## INTRODUCTION

**FIG 1:**
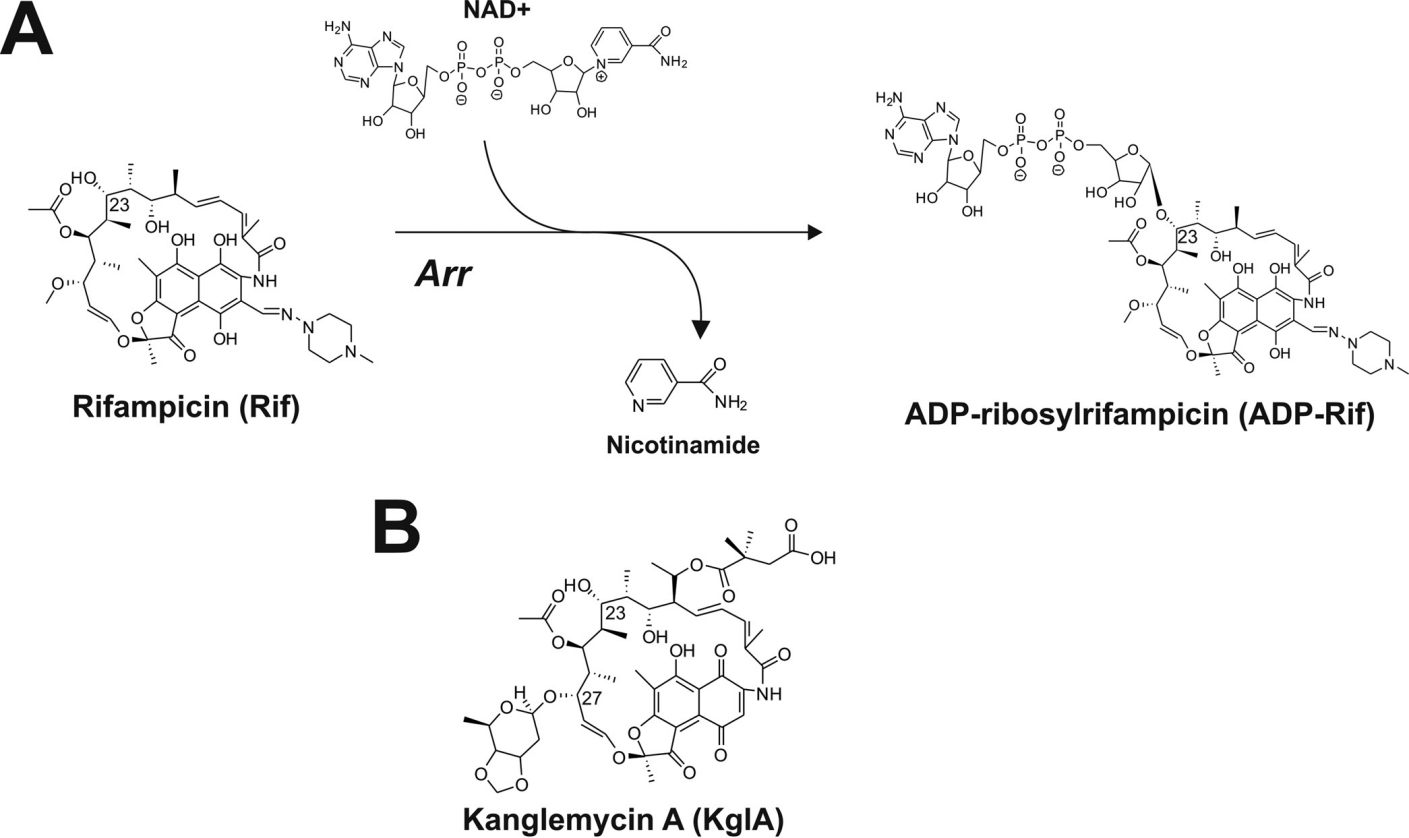
(A) ADP-ribosylation by rifampicin-ADP-ribosyl transferases (Arr) of rifampicin (Rif). (B) Structure of KglA.

Resistance to the front-line drug used in treatment of tuberculosis, rifampicin (Rif), is typically conferred through point mutations within the *rpoB* gene encoding the Rif-binding β-subunit of RNA polymerase (RNAP) (1). However, Rif is also subject to enzymatic inactivation by several recently described bacterial enzymes (2–5). ADP-ribosylation of Rif by Mycobacterium smegmatis rifampicin ADP-ribosyl transferase (Arr_Ms_) is believed to preclude Rif binding to the RNAP β subunit, although this has not yet been directly demonstrated. Arr_Ms_ utilizes NAD^+^ to catalyze the ADP-ribosylation of Rif at the C-23 hydroxyl group, with concurrent release of the nicotinamide moiety from NAD^+^ (6) (Fig. 1A). Rifamycin SV, and the newer semisynthetic Rif derivatives rifaxamin and rifabutin, are also substrates for Arr_Ms_, suggesting the enzyme has a broad substrate specificity for rifamycins (3, 7). However, it has been shown that C-25 carbamate derivatives of Rif exhibit greatly improved antimicrobial activity against M. smegmatis (8). *In vitro* experiments with purified Arr_Ms_ indicated that C-25 carbamate rifamycins are resistant to modification by Arr (8). The recently solved crystal structure of Arr_Ms_ in complex with Rif provides a rational explanation for this evasion of Arr inactivation; the supposed position of the C-25 carbamate group would afford a pronounced clash within the Arr_Ms_ Rif-binding pocket (3).

The emerging pathogen Mycobacterium abscessus is one of the most clinically important nontuberculous mycobacteria that are responsible for severe respiratory, skin, and mucosal infections in humans. Because of resistance to most classes of antibiotics, including rifamycins, infections caused by M. abscessus remain extremely difficult to treat (9). The genome of M. abscessus encodes a homologue of Arr_Ms_ (Arr_Mab_) that confers innate high-level Rif resistance. Deletion of Arr_Mab_ significantly increases not only the sensitivity of M. abscessus to Rif but also that to other rifamycins, i.e., rifabutin, rifaximin, and rifapentine (7, 10, 11). Therefore, rifamycins that could evade the activity of Arr_Mab_ could be promising treatment candidates. The C-25-modified rifamycins have, similarly to Rif, high MICs against wild-type M. abscessus but considerably lower MICs against a mutant lacking Arr_Mab_ (11), suggesting that the putative Arr_Mab_ may have a different substrate specificity to Arr_Ms_.

The natural product kanglemycin A (KglA) is an ansamycin antibiotic that, like other rifamycins, inhibits transcription by binding within the rifampicin-binding pocket on the β-subunit of RNAP (12). However, KglA has unique substituents present on the *ansa* bridge, namely, a 2,2-dimethylsuccinic acid chain at C-20 and a β-*O*-3,4-*O,O*′ methylene digitoxose moiety at C-27. When bound to RNAP, these substituents afford additional binding contacts in the rifampicin-binding pocket and produce an altered binding conformation that leads to overcoming rifampicin-resistant amino acid substitutions (12).

We hypothesized that these large, bulky substituents may also prevent KglA binding to Arr or that the ADP-ribosylated KglA may be able to bind to RNAP due to its different mode of binding compared to that of Rif. In this study, we show that M. smegmatis Arr indeed cannot bind and modify KglA. In contrast, a homologue of Arr_Ms_ from M. abscessus, which we show is indeed a functional Rif ADP-ribosyl transferase, binds and modifies KglA with the same efficiency as that of Rif. We also, for the first time, directly show that ADP-ribosylated Rif does not inhibit bacterial RNAP.

## RESULTS

### M. smegmatis Arr (Arr_Ms_) ADP-ribosylates Rif but not KglA.

**FIG 2:**
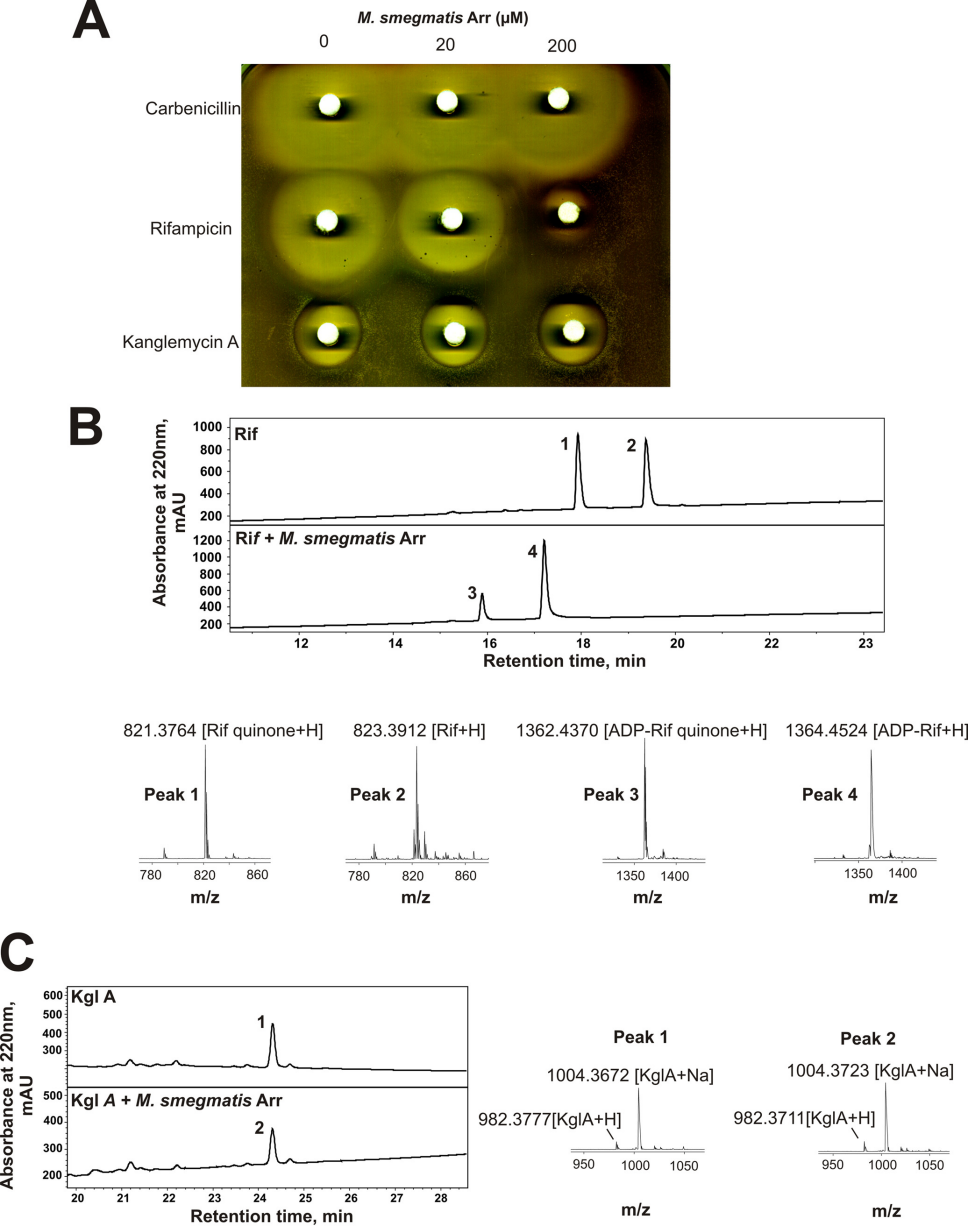
M. smegmatis Arr (Arr_Ms_) fails to inactivate kanglemycin A (KglA) by ADP-ribosylation. (A) Disk diffusion assay of aliquots of *in vitro* reaction mixtures with 1 mg/ml carbenicillin, rifampicin, or kanglemycin A incubated in the presence of indicated concentrations of Arr_Ms_ in 10-μl reaction mixtures. Reaction mixtures were transferred onto the paper disks, which were placed on LB agar plates infused with a lawn of S. aureus RN4220 (8). (B) Reversed-phase high-performance liquid chromatography (HPLC) traces of *in vitro* modification of rifampicin (Rif) by Arr_Ms._ Plots show Rif incubated with NAD^+^ in the absence (upper trace) or presence (lower trace) of Arr_Ms_. Below are the positive ion mass spectra of peaks 1, 2, 3, and 4. Ion adducts are as indicated. (C) Reversed-phase HPLC traces of KglA incubated with NAD^+^ and with or without Arr_Ms_, and mass spectra of peaks 1 and 2. Note that Rif and KglA have different absorbance spectra, and the areas or the heights of their chromatography peaks cannot be directly compared.

In order to characterize Arr_Ms_ *in vitro*, we cloned the Arr gene from M. smegmatis into a pET28 expression vector and expressed and purified the protein from Escherichia coli. To investigate the inactivation of antibiotics by Arr_Ms_, we performed *in vitro* inactivation reactions with a tandem disk assay (Fig. 2A). Increasing concentrations of Arr_Ms_ were incubated with NAD^+^ and antibiotic and then spotted onto paper disks which were placed onto a lawn of Staphylococcus aureus RN4220. Apparent decreases in the sizes of the zones of inhibition were interpreted as functional inactivation of the antibiotic. As shown in Fig. 2A, the control antibiotic carbenicillin is not inactivated by Arr_Ms_. Rif is inactivated by Arr_Ms_, resulting in decreased sizes of zones of inhibition when Arr_Ms_ concentration is increased. However, KglA appears to be resistant to inactivation by Arr_Ms_, as the size of the zone of inhibition does not decrease even at very high concentrations of Arr_Ms_. The results of the disc assay were corroborated by measurement of MIC for the drugs treated with Arr_Ms_ or left untreated (Table 1). Note that the smaller zones of inhibition and higher MICs seen with KglA, compared to those with Rif, is thought to be due to poorer penetration of the compound through the cell envelope. These data indicate that KglA is not a substrate for Arr_Ms_.

**TABLE 1:** MICs of Rif and KglA treated with Arr_Sm_ and Arr_Mab_ enzymes

Drug	MIC (μg/ml) of drug with:		
	No treatment	Treatment with M. smegmatis Arr	Treatment with M. abscessus Arr
Rif	0.007	>125	>125
KglA	0.488	0.488	>125

To directly analyze ADP-ribosylation by Arr_Ms_, we utilized a tandem liquid chromatography-mass spectrometry (LC-MS)-based assay in which antibiotic and NAD^+^ substrates were incubated in the absence or presence of Arr_Ms_ and then the reaction products were separated by high-performance liquid chromatography (HPLC) and identified by mass spectrometry. When Rif is incubated with NAD^+^ in the absence of Arr_Ms_, Rif and its oxidized form rifampicin quinone (Rifq) are resolved with no identifiable ADP-ribosylated product (Fig. 2B). This indicates that the ADP ribosylation reaction requires enzymatic catalysis. Indeed, when incubated in the presence of NAD^+^ and Arr_Ms_, both Rif and Rifq are ADP-ribosylated, confirming the activity of purified Arr_Ms_ (Fig. 2B). Interestingly, however, incubation of KglA with NAD^+^ and Arr_Ms_ failed to ADP-ribosylate KglA, indicating that KglA is not a substrate of Arr_Ms_ (Fig. 2C), which is consistent with results of the above-described *in vivo* assay.

### M. abscessus Arr (Arr_Mab_) ADP-ribosylates both Rif and KglA.

**FIG 3:**
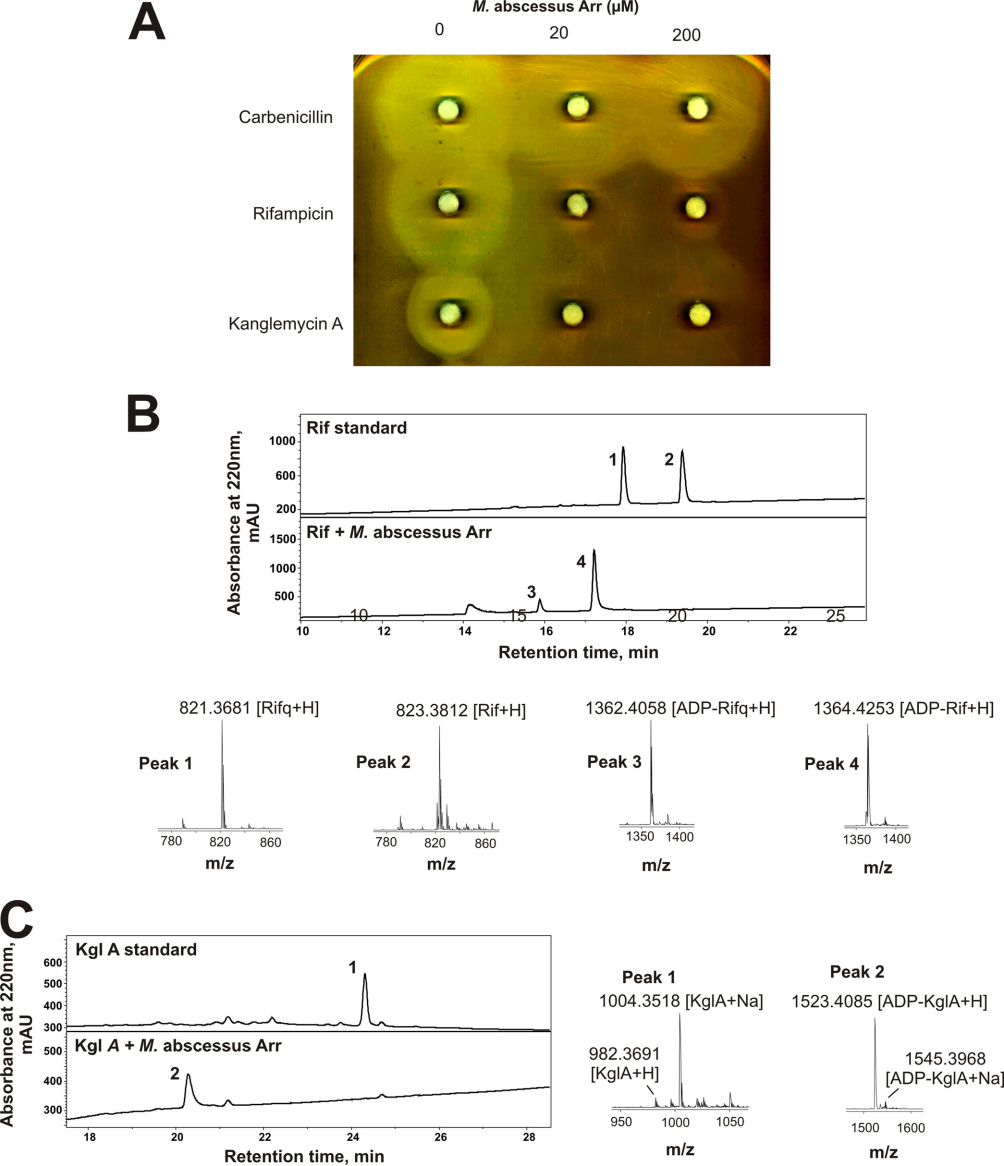
M. abscessus Arr (Arr_Mab_) inactivates kanglemycin A (KglA) by ADP-ribosylation. (A) Disk diffusion assay of aliquots of *in vitro* reaction mixtures with 1 mg/ml carbenicillin, rifampicin, or kanglemycin A incubated in the presence of the indicated concentrations of Arr_Mab_ in 10-μl reaction mixtures. Reaction mixtures were transferred onto the paper disks, which were placed on LB agar plates infused with a lawn of S. aureus RN4220. (B) Reversed-phase HPLC traces of *in vitro* modification of rifampicin (Rif) by Arr_Mab._ Plots show Rif incubated with NAD^+^ in the absence (upper trace) or presence (lower trace) of Arr_Mab_. Below are the positive ion mass spectra of peaks 1, 2, 3, and 4. Ion adducts are as indicated. (C) Reversed-phase HPLC traces of KglA incubated with NAD^+^ and with or without Arr_Mab_, and mass spectra of peaks 1 and 2.

Prior genetic experiments have indicated the substrate specificity of a putative ADP-ribosyl transferase from M. abscessus, Arr_Mab_, may differ from that of Arr_Ms_ (11). Therefore, we aimed to determine the function and activity of Arr_Mab_ *in vitro*. First, a purified Arr_Mab_ was analyzed in the disk assay, as described above. As can be seen from Fig. 3A (middle row), Rif activity is almost completely eliminated by both concentrations of Arr_Mab_, as judged by disappearance of the zone of inhibition when Rif is incubated with Arr_Mab_. Unexpectedly, unlike Arr_Ms_, Arr_Mab_ was also capable of inactivating KglA (Fig. 3A, bottom row). Measurement of MIC confirmed this observation (Table 1).

To resolve this apparent controversy, we directly analyzed activity of Arr_Mab_ using a tandem LC-MS-based assay, as we did for Arr_Ms_. Incubation of Rif and Rifq with NAD^+^ and Arr_Mab_ resulted in their ADP-ribosylation (Fig. 3B). However, unlike in the case with Arr_Ms_, incubation of KglA with Arr_Mab_ and NAD^+^ also resulted in ADP-ribosylation of the antibiotic (the exact mass of the product was 1,523.40 Da, the predicted mass of ADP-ribosyl KglA; Fig. 3C). This finding corroborates the results of the above-described disk assay. The results also confirm that the homologue of Arr_Ms_ from M. abscessus indeed encodes a functional rifamycin ADP-ribosyl transferase that has broader substrate specificity than that of Arr_Ms_.

### Rif and KglA binding affinities at Arr_Mab_ and Arr_Ms_.

**FIG 4:**
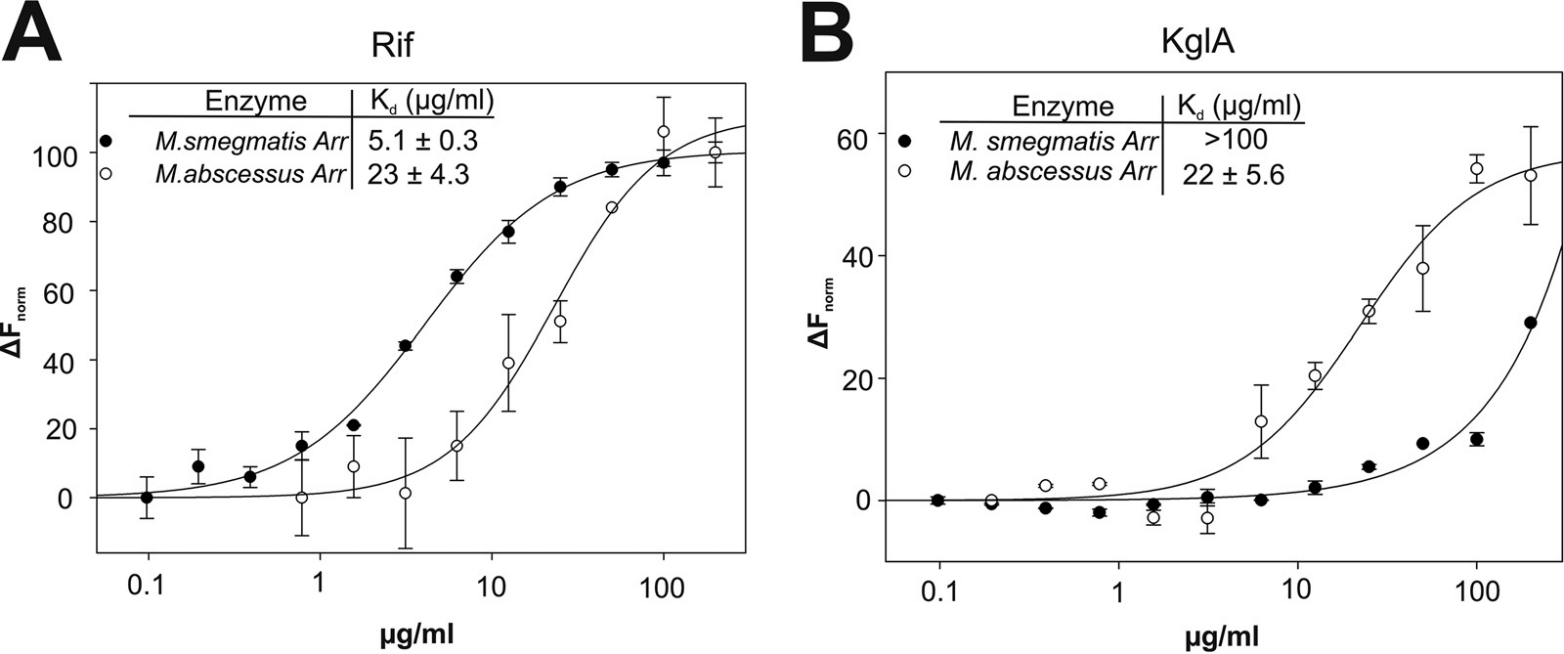
Substrate specificity of Arr_Ms_ and Arr_Mab_. Binding affinities of Rif (A) and KglA (B) at labeled Arr_Ms_ and Arr_Mab_, identified by microscale thermophoresis. Normalized fluorescence (*F*_norm_; fluorescence after thermophoresis/initial fluorescence) is plotted against antibiotic concentration. Error bars ± standard deviation (SD) are shown.

The inability of Arr_Ms_ to ADP-ribosylate KglA can be explained by either inability to bind the KglA molecule or inability to perform the catalysis. To distinguish between these possibilities, we investigated the binding of Rif and KglA to Arr_Ms_ and Arr_Mab_ using microscale thermophoresis (MST). Apparent dissociation constants (*K_d_*) were determined by titrating serial dilutions of antibiotics against Arr_Ms_ and Arr_Mab_. The affinity of Arr_Ms_ to KglA was much lower than its affinity to Rif (Fig. 4A and B), suggesting that the bulky 2,2-dimethylsuccinic acid chain at C-20 and/or β-*O*-3,4-*O,O*′ methylene digitoxose at C-27 of KglA prevents binding of the compound to the Arr_Ms_ active site. In contrast, Arr_Mab_ bound both antibiotics with the same *K_d_*. The result indicates that the inactivity of Arr_Ms_ toward KglA is explained by the reduced binding affinity to the antibiotic. Notably, Rif binds with greater affinity to Arr_Ms_ than to Arr_Mab_ (Fig. 4A), suggesting that the broader substrate specificity of Arr_Mab_ comes at a cost of reduced binding affinity.

### ADP-ribosylation renders rifamycins inactive against RNA polymerase.

**FIG 5:**
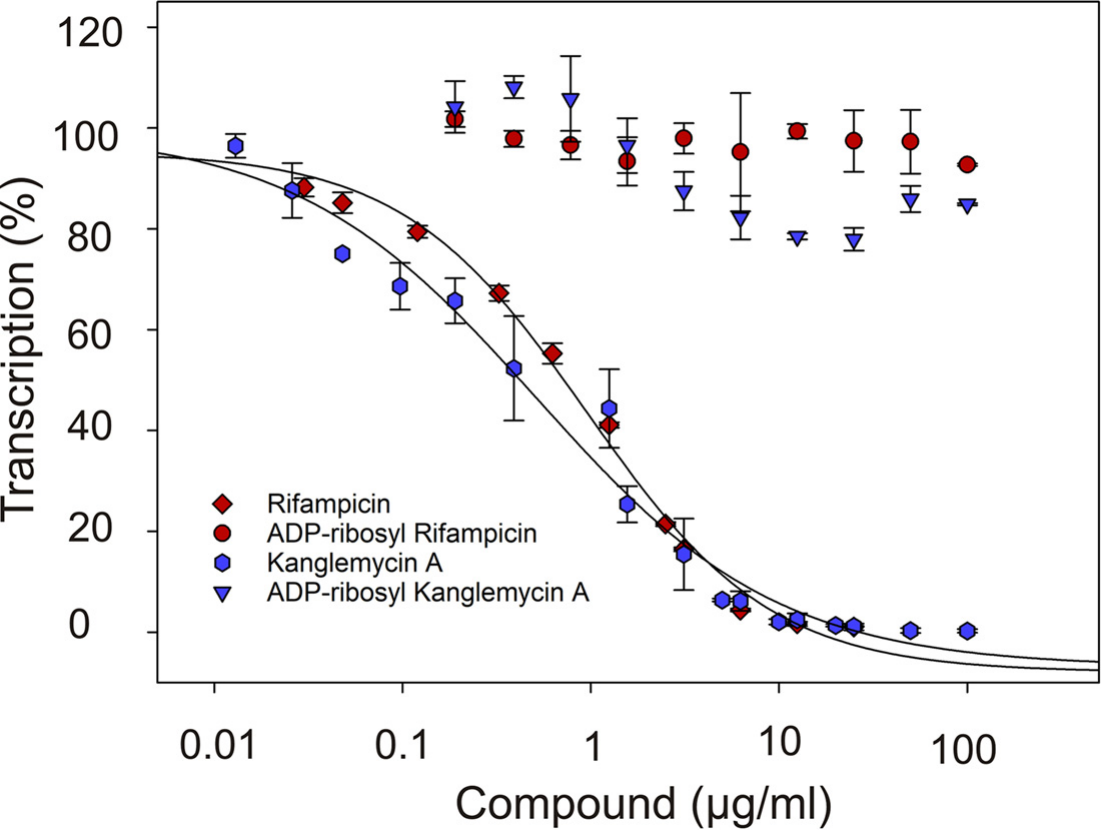
ADP-ribosylated rifamycins fail to inhibit RNAP. Effects of Rif and KglA and ADP-ribosylated Rif and KglA on *in vitro* transcription by E. coli RNAP on a linear DNA template containing the T7A1 promoter. Runoff and terminated transcription products were normalized to those synthesized in the absence of inhibitor. Error bars ± SD are shown.

It is not known how ADP-ribosylation of rifamycins by Arr enzymes inactivates the antibiotics. Therefore, we purified ADP-ribosyl-Rif and ADP-ribosyl-KglA from the reactions catalyzed by Arr_Ms_ and Arr_Mab_, respectively, and analyzed their effects on *in vitro* transcription by E. coli RNAP on a linear DNA template containing the T7A1 promoter. As can be seen in Fig. 5, consistently with our previous study (12), both Rif and KglA efficiently inhibited transcription with submicromolar 50% inhibitory concentrations (IC_50_). In contrast, ADP-ribosyl-Rif and ADP-ribosyl-KglA were inactive even at high concentrations (100 μM). Because part of the mechanism of inhibition by all rifamycins is the sterical occlusion of the pathway of the nascent RNA (13), this result suggests that ADP-ribosylation prevents rifamycins binding to RNAP.

## DISCUSSION

Rifamycins inhibit bacterial transcription by targeting the β-subunit of RNAP (13). One of the findings of this work is that ADP-ribosylation of Rif by Arr proteins completely abolishes its activity against RNAP *in vitro*. Addition of the ADP-ribosyl at C-23 abolishes a critical hydrogen bond between the Rif molecule and the RNAP and also orientates the bulky substituent toward the surface of the Rif-binding pocket on RNAP, likely causing a severe steric clash.

**FIG 6:**
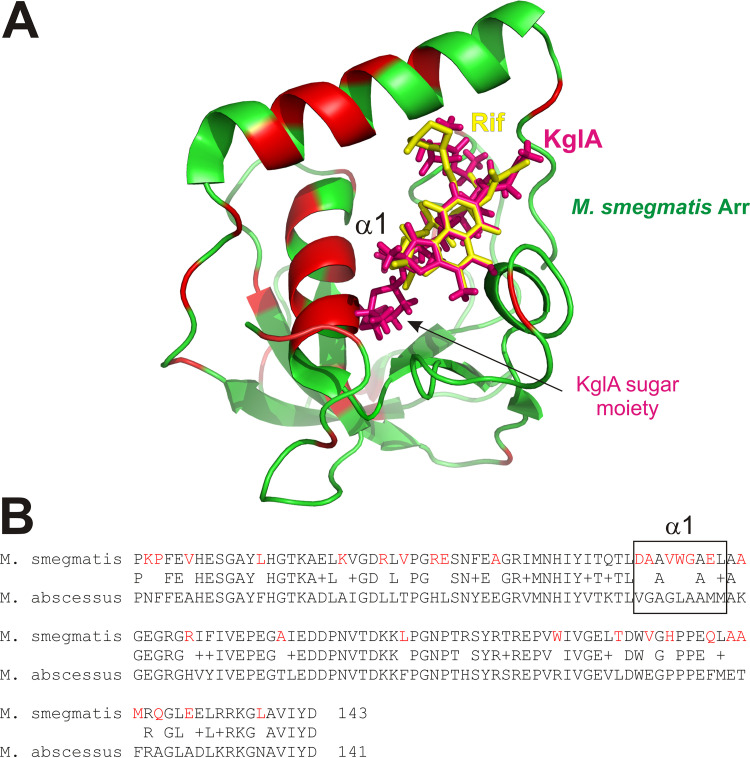
(A) Structural alignment of the KglA molecule (magenta) and Rif (yellow) complexed with M. smegmatis Arr (green; PDB identifier 2HW2) (3). Red patches show absence of homology with Arr_Mab._ (B) Sequence alignment of Arr_Ms_ and Arr_Mab_. Alpha helix 1 (α1) is highlighted.

Importantly, however, we show that KglA, a rifamycin with unique bulky substituent present on the *ansa* bridge, is not a substrate for Arr_Ms_, as a consequence of reduced binding affinity to the enzyme. If KglA adopts a similar conformation to that of Rif within the Rif-binding pocket of Arr_Ms_, a strong steric clash occurs between the C-27 β-*O*-3,4-*O,O*′ methylene digitoxose moiety of KglA and an α-helix (residues 54 to 65) that constitutes one half of the Rif-binding cleft of Arr_Ms_ (Fig. 6A). This α-helix, termed α1, is implicated in Rif-binding interactions; residue D55 makes polar interactions with O-11 of Rif, while residues A56, W59, G60, and L63 form nonpolar interactions with the carbon backbone of the *ansa* bridge (3). Arr homologues are widely distributed across various pathogenic and environmental bacteria (E. coli, Pseudomonas aeruginosa, Klebsiella pneumoniae, Acinetobacter baumannii, Stenotrophomonas maltophilia, Burkholderia cenocepacia, many anaerobic bacteria, and different actinomycetes species, such as Streptomyces coelicolor). Considering that KglA also retains activity against Rif-resistant RNAP and bacteria (12, 14), the antibiotic appears to be a promising lead compound with which to target some (but not all) bacteria possessing Arr enzymes.

We also characterized a homologue of Arr_Ms_ encoded by the M. abscessus genome and confirmed that it is a true ADP-ribosylase of Rif. Like Arr_Ms_, Arr_Mab_ utilizes NAD^+^ to catalyze the ADP-ribosylation of Rif. However, unlike Arr_Ms_, KglA remains a substrate for ADP-ribosylation by Arr_Mab_. The amino acid sequences between Arr_Ms_ and Arr_Mab_ are very similar, exhibiting a 66% sequence identity and 76% homology (Fig. 6B). However, Arr_Mab_ α1 bears almost no sequence similarity with that of Arr_Ms_. This lack of homology in α1 may underlie the broader substrate specificity exhibited by Arr_Mab_. However, the variation of α1 residues involved in Rif binding may also explain the reduced affinity with which Arr_Mab_ binds Rif (Fig. 4).

There is strong genomic evidence that most innate resistance mechanisms seen in clinical pathogens are of environmental origin (15). The often-communal existence of bacteria generates pressure to gain a competitive edge over neighboring microorganisms. Consequently, bacteria have evolved a number of mechanisms to counter antibiotics secreted by other microbes in their surroundings. Bulky ansamycin compounds, such as KglA, are produced by actinomycetes that occupy the same natural niches as nontuberculous mycobacteria, such as M. abscessus (14, 16). It is tempting to speculate that in its evolution, M. abscessus has encountered KglA or a similar natural rifamycin with a modified *ansa* bridge.

It should be noted that the precise physiological role of Arr enzymes remains unclear, as Arr has been shown to play roles in cellular pathways even in the absence of rifamycins. For example, Arr is upregulated in response to different stresses (e.g., double-stranded DNA breaks, starvation, oxidative stress, and ciprofloxacin treatment) (17). In M. smegmatis, Arr contributes to biofilm formation and confers a growth fitness advantage. Although its expression has been shown to be associated with the stringent response, the mechanism of stress-correlated gene induction is unclear (18). It is possible that the main function of Arr is still inactivation of rifamycins, but the presence of rifamycins causes similar cell stresses (such as double-strand breaks caused by collisions of RNAP with replication machinery) that signal for production of Arr.

## MATERIALS AND METHODS

### Reagents, antibiotics, and DNA templates.

All chemicals, reagents, and antibiotics were purchased from Sigma unless otherwise stated. The T7A1 promoter fragment was produced by PCR with the primers 5′-CGACGTTGTAAAACGACGGCCAGTG-3′ and 5′-GGTCGACTCTAGAGGATCGCT-3′ (IDT) from the template GGTCGACTCTAGAGGATCGCTATAACAGGCCTGCTGGTAATCGCAGGCCTTTTTATTTGGATCCAGATCCCGAAAATTTATCAAAAAGAGTATTGACTTAAAGTCTAACCTATAGGATACTTACAGCCATCGAGAGGGACACGGCGAATAGCCATCCCAATCGACACCGGGGTCCGGGATCTGGATCTGGATCGCTAATAACAGGCCTGCTGGTAATCGCAGGCCTTTTTATTTGGATCCCCGGGTACCGAGCTCGAATTCACTGGCCGTCGTTTTACAACGTCG and was purified by agarose gel electrophoresis. Kanglemycin A was purified as described previously (12).

### Protein expression and purification.

E. coli RNAP core and σ^70^ were purified exactly as described previously (19). M. smegmatis and M. abscessus Arr were expressed in T7 express cells (New England Biolabs) transformed with pET28 expression vector encoding N-terminal 6×His-tagged M. smegmatis Arr or M. abscessus Arr. Expression was induced with 0.4 mM isopropyl-β-d-thiogalactopyranoside (IPTG) in exponentially growing cells, which were then incubated overnight at room temperature on an orbital shaker (150 rpm). Cells were then harvested by centrifugation and resuspended in grinding buffer (50 mm Tris-HCl [pH 7.9], 10% glycerol, 200 mM NaCl, and protease inhibitor mixture [Roche]). Cells were then lysed by sonication and debris cleared by centrifugation. Arr enzymes were then purified by HisTrap HP (Cytiva) nickel affinity chromatography, concentrated, and dialyzed into storage buffer (50 mM Tris-HCl [pH 7.9], 50% glycerol, 200 mM NaCl, and 2 mM β-mercaptoethanol).

### *In vitro* rifampicin ADP-ribosyl transferase activity assay.

Reactions were performed in a 100-μl final volume of Arr buffer (20 mM Tris-HCl [pH 7.9], 40 mM KCl, and 0.5 mM MgCl_2_). M. smegmatis or M. abscessus Arr (10 μM) was mixed with Rif or KglA (100 μM) in 80 μl of Arr buffer at 37°C for 10 min. A 20-μl aliquot of NAD^+^ in Arr buffer was added (250 μM final) and incubated for 1 h at 37°C. The reaction was quenched with 500 μl of methanol (MeOH). Methanol was then evaporated under negative pressure and the reaction mixture analyzed by LC-MS. All analytical separations were performed on an Agilent 1260 HPLC instrument by injection of 1 to 5 μl of sample onto a Raptor ARC-18 column (150 mm by 2.1 mm) (Restek) or an Ultra C_4_ column (150 mm by 2.1 mm) operated at 0.2 μl/min and then eluted using a 30-min linear gradient from 5% to 100% of acetonitrile. The mobile phase was supplemented with 0.1% formic acid. Mass spectra were recorded in positive-ion mode on a Bruker MicrOTOF II time-of-flight mass spectrometer.

### Rifampicin ADP-ribosyl transferase disk assay.

Reactions were performed in a 10-μl final volume of Arr buffer. M. smegmatis or M. abscessus Arr at an indicated concentration (0 μM, 20 μM, or 200 μM) was mixed with antibiotic (1 mg/ml final) in 8 μl Arr buffer at 37°C for 5 min. A 2-μl aliquot of NAD^+^ in water was added (10 mM final) and incubated for 1 h at 37°C. The reaction was quenched with an equal volume of methanol, the mixture spotted onto paper disks, and a disk assay performed with an embedded lawn of S. aureus RN4220 as described previously (20).

### Rifampicin ADP-ribosyl transferase MIC assay.

MICs were quantified by broth microdilution assay using the S. aureus RN4220 strain at a starting cell density of 2 × 10^5^ to 5 × 10^5^ CFU/ml and tryptic soy broth (Oxoid). The compounds were dissolved in dimethyl sulfoxide (DMSO). Cultures were incubated for 24 h at 37°C, and MIC was recorded as the lowest concentration of each drug leading to inhibition of visible growth. Reproducibility was ensured by repeating all tests at least twice on separate occasions.

### Purification of ADP-ribosyl rifampicin.

The reaction was performed in a 2,000-μl volume of Arr buffer containing 25 μM M. smegmatis Arr, 5 mg rifampicin, and 20 mM NAD^+^. The reaction mixture was incubated at 37°C for 24 h and cleaned up on a 25 ml HyperSep C_8_ solid-phase extraction (SPE) cartridge. ADP-ribosylated Rif was eluted with 30% MeOH and dried under negative pressure to yield 4.8 mg of ADP-ribosyl-Rif. Sample homogeneity was confirmed by tandem LC-MS, as described above.

### *In vitro* transcription.

Transcription from promoter DNA fragments was performed essentially as described previously (12, 19). Reactions were performed in 10 μl of transcription buffer TB (20 mM Tris HCl [pH 7.9], 40 mM KCl, and 10 mM MgCl_2_) containing 1 pmol of coli RNAP core with 3 pmols of σ^70^ and 10% DMSO with or without inhibitors. Transcription was initiated by the addition of a mixture of 25 μM CpA dinucleotide as a primer, 0.2 μl α-[^32^P] UTP (10 mCi/ml; Hartmann Analytic), 10 μM UTP, 100 μM ATP, 100 μM CTP, and 100 μM GTP, and 10 nM promoter DNA. Reactions were stopped after 10-min incubation at 37°C by the addition of equal volume of formamide-containing loading buffer. Products were resolved in denaturing polyacrylamide gels, revealed by phosphorimaging (Cytiva), and analyzed using ImageQuant software (Cytiva).

### Microscale thermophoresis.

Binding affinity experiments were carried out on a Monolith NT.115 series instrument (Nano Temper Technologies GMBH). M. smegmatis and M. abscessus Arr were labeled with Monolith Protein labeling kit RED-NHS second-generation amine (Nano Temper Technologies GMBH) according to the manufacturer’s guidelines. Roughly 5 μl of sample in MST buffer (20 mM HEPES [pH 7.9], 40 mM KCl, and 10 mM MgCl_2_) were loaded into Monolith NT.115 Premium capillaries, and thermophoresis was measured for 30 s. Analysis was performed with Monolith software. *K_d_* was quantified by analyzing the change in normalized fluorescence (*F*_norm_; fluorescence after thermophoresis/initial fluorescence) as a function of inhibitor concentration. Curves for *K_d_* data were fitted to a 4-parameter logistic equation using nonlinear regression in SigmaPlot software.
